# Effects of donor-specific microvascular anatomy on hemodynamic perfusion in human choriocapillaris

**DOI:** 10.1038/s41598-023-48631-2

**Published:** 2023-12-19

**Authors:** Senyou An, Huidan Yu, MD Mahfuzul Islam, Xiaoyu Zhang, Yuting Zhan, Joseph J. Olivieri, Jayakrishna Ambati, Jun Yao, Bradley D. Gelfand

**Affiliations:** 1 State Key Laboratory of Intelligent Construction and Healthy Operation and Maintenance of Deep Underground Engineering, Shenzhen University, Shenzhen, 518060 China; 2 Department of Mechanical and Energy Engineering, Indiana University-Purdue University, Indianapolis, IN 46202 USA; 3 Department of Vascular Surgery, Indiana University School of Medicine, Indianapolis, IN 46202 USA; 4 Dyson School of Design Engineering, Imperial College London, London, SW7 2AZ UK; 5 Center for Advanced Vision Science, University of Virginia School of Medicine, Street, Charlottesville, VA 22908 USA; 6 Department of Pathology, University of Virginia School of Medicine, Street, Charlottesville, VA 22908 USA; 7 Department of Ophthalmology, University of Virginia School of Medicine, Street, Charlottesville, VA 22908 USA; 8 Department of Microbiology, Immunology, and Cancer Biology, University of Virginia School of Medicine, Street, Charlottesville, VA 22908 USA; 9 Research Center of Multiphase Flow in Porous Media, China University of Petroleum (East China), Qingdao, 266580 China; 10 Department of Biomedical Engineering, University of Virginia School of Medicine, Street, Charlottesville, VA 22908 USA

**Keywords:** Translational research, Biotechnology, Anatomy, Pathogenesis, Diseases, Eye diseases, Macular degeneration

## Abstract

Evidence from histopathology and clinical imaging suggest that choroidal anatomy and hemodynamic perfusion are among the earliest changes in retinal diseases such as age-related macular degeneration (AMD). However, how inner choroidal anatomy affects hemodynamic perfusion is not well understood. Therefore, we sought to understand the influences of choroidal microvascular architecture on the spatial distribution of hemodynamic parameters in choriocapillaris from human donor eyes using image-based computational hemodynamic (ICH) simulations. We subjected image-based inner choroid reconstructions from eight human donor eyes to ICH simulation using a kinetic-based volumetric lattice Boltzmann method to compute hemodynamic distributions of velocity, pressure, and endothelial shear stress. Here, we demonstrate that anatomic parameters, including arteriolar and venular arrangements and intercapillary pillar density and distribution exert profound influences on inner choroidal hemodynamic characteristics. Reductions in capillary, arteriolar, and venular density not only reduce the overall blood velocity within choriocapillaris, but also substantially increase its spatial heterogeneity. These first-ever findings improve understanding of how choroidal anatomy affects hemodynamics and may contribute to pathogenesis of retinal diseases such as AMD.

## Introduction

The choroid is an anatomically unique vascular network that supports the nutrient exchange of the photoreceptor and retinal pigmented epithelial (RPE) layers of the retina. The inner choroid is comprised of arterioles and venules from Sattler’s layer that feed and drain the choriocapillaris, which is a highly anastomotic planar capillary bed. Because phototransduction and the visual cycle are extremely metabolically demanding, the arterioles of the macular choroid subsume among the greatest blood flow of the entire body per unit mass^1^. Beyond metabolic transport, the choroid is also responsible for, among other functions, trafficking of circulating and resident inflammatory cells to sites of infection, damage, or disease of the outer retina^2–7^. Failure of the choroid to fulfil its diverse functions can contribute to retinal disease.

Vascular remodelling of the choroid through neovascularization or atrophy is a common etiologic element of numerous blinding ocular conditions, including age-related macular degeneration (AMD), diabetic retinopathy, and glaucoma. For example, histopathologic analyses of human donor eyes reveals loss of capillary density and diameter in AMD^8–10^, and capillary involution that may extend beyond the margin of retinal death in late AMD^11^.

These histopathologic observations have been supported by functional imaging of choroidal perfusion via indocyanine green angiography (ICGA), optical coherence tomography angiography (OCTA), and laser Doppler flowmetry (LDF), which have detected alterations in choroidal blood flow rate^12–14^ and focal hypoperfusion^15,16^ in patients with AMD. Indeed, emerging evidence suggests that such perfusion alterations also extend beyond the margins of retinal atrophy and may predict disease progression^17–22^.

Given observations of choroidal microvascular anatomic changes in aging and in retinal diseases in cadaver eyes, coupled with those from functional perfusion imaging in living patients, we and others have sought to connect these two domains of knowledge by understanding the relationship between microvascular anatomy and inner choroidal blood flow.

Efforts to describe inner choroidal hemodynamics have applied fluid dynamics principles. The choriocapillaris has previously been treated as a thin plane of uniform thickness interrupted by intercapillary pillars modeled either as uniform cylinders^23^, porous media^24^, or an open plane^25,26^. In these geometries, blood is supplied and drained by cylindrical and regularly spaced arterioles and venules^23,24^, respectively. While prior studies have improved the understanding of the general properties of blood flow in the inner choroid, the extent to which these findings are valid in anatomically realistic human choroidal vasculature is unknown. Moreover, the precise influence of aging and disease-associated anatomic changes in choroidal microvasculature on perfusion are not known.

In addition, the presence of functional lobules or vascular segments in inner choroid have been observed in monkeys using fluorescent dyes^27,28^, and in the simplified model developed by Ref.^25^. However the nature of these lobules has remained controversial because of the lack of obvious anatomic evidence for such independent units in humans^29^.

To address these knowledge gaps, we employed a unique imaged-based computational hemodynamics (ICH) approach, InVascular^30–32^, to study hemodynamics in 3-dimensional (3-D) human choriocapillaris extracted from the images of high-resolution fluorescent confocal microscopy of human donor eyes. Briefly, InVascular is our in-house computational platform integrating volumetric lattice Boltzman method (VLBM)^30^ with GPU (graphics processing unit) parallel computing. It has been used effectively and accurately to compute fluid dynamics in arbitrarily complicated flow domains. The kinetic-based lattice Boltzmann method (LBM) is a class of CFD(computational fluid dynamics) methods for simulating complex flows including pore-scale porous media flows^33,34^, multiphase/multicomponent flows^35,36^, and turbulence^37,38^. Using InVascular, we acquired 3-D distributions of velocity, pressure, and endothelial shear stress (ESS) in the inner choroids of 8 donor eye cases for hemodynamic characteristics. We then examined the influences of choriocapillary structure and arteriole/venule distribution on inner choroid hemodynamics by modulating capillary morphology and arteriole/venular distribution, respectively, and establishing the relationship between anatomy and blood flow pattern in the inner choroid.

## Results

### Hemodynamic characteristics in choriocapillaris of donor eye cases

**Figure 1:**
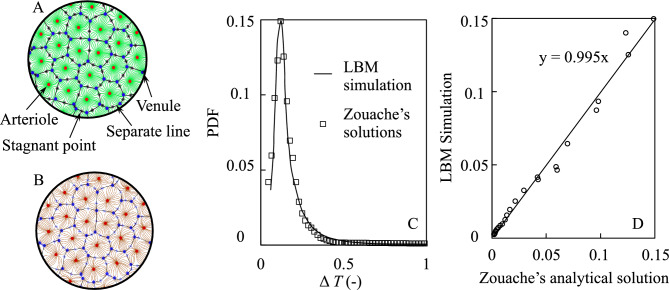
Comparisons of flow pattern between the InVascular simulation results and a prior analysis^25^. (**A**,**B**) The streamlines, separation lines, and stagnation points of blood flow in the mid-plane of the choriocapillaris from Zouache et al.^25^ (**A**) and from the present InVascular simulation (**B**). (**C**) The PDF of the travel time of a corpuscle calculated for ten adjacent functional vascular segments of a randomly generated distribution of arteriolar and venular openings. (**D**) The correlation of PDFs shows agreement between the two. Pearson’s R = 0.9921, P $<10^{-5}$.

We first sought to validate InVascular by analyzing blood flow in a functional vascular segment of choriocapillaris inspired by imaging data of primate inner choroid^25^, for which the blood flow pattern and probability density function (PDF) of corpuscle travel time have been previously determined. As shown in Fig. 1A, arterioles (red) and venules (blue) are placed on an inner choroid layer with a quasi-random distribution within a predefined number of vascular segments, which were defined by a prespecified number of arterioles and venules. An empty center plane was used for modeling the choriocapillaris. Blood flow is driven by a pressure difference between the arterioles (red dots) and venules (blue dots). Zouache et al.^25^ revealed the flow field with green streamlines as shown in Fig. 1A. The separation surfaces of the flow field, which delineate functional vascular segments, are plotted as a series of connected blue lines. Stagnation points are represented as asterisks. Our InVascular simulation agreed well with this published flow field (Fig. 1B). We also compared the PDF of the travel time of corpuscles between the InVascular simulation result (line) and the published analytical solution (dots) (Fig. 1C). The travel time was defined as the time a particle enters an arteriole and exits through a venule. The PDF was calculated for ten adjacent functional vascular segments as in Zouache et al.^25^. Figure 1D presents the correlation of the PDF between Zouache’s and our simulation results. Quantitatively good agreement demonstrates the reliability of InVascular for revealing the hemodynamics in the inner choroidal flow domain.

Next, we applied InVascular to anatomies extracted from the inner choroid regions dissected from the inner choroid regions dissected from the posterior pole of eight human cadaver eyes. In brief, human donor eyes were acquired from which the inner choroid was isolated, fluorescently labelled to delineate vascular structures, and imaged in three dimensions. The vascular network was then segmented by identifying capillaries and arteriole and venular junctions. A periodic boundary condition was applied to both sides of the layers and the walls of the choriocapillaris, arterioles, and venules were rigid. Blood density, of 1060 kg/m$^3$ and kinematic viscosity of $3.8\times 10^{-6}$ m$^2$/s, were used to model blood. This network was then subjected to ICH to obtain velocity vector, pressure, and ESS, a key biomechanical signal governing endothelial cell physiology^39^. Finally, the 3-D hemodynamic fields were interpreted via post-processing. We used a pilot case to iteratively determine that a constant pressure gradient between the inlets of arterioles and venules of 44.2 mmHg and 35.6 mmHg respectively, resulted in average blood velocity of 1.5 mm/s, which is within physiologic range of choriocapillaris blood velocity measured in living non-human primate subjects via fluorescent dye tracing^40^, leukocyte tracing^41^, and in healthy human subjects via fluorescent dye injection^42^. Descriptive statistics of anatomic parameters from the eight cases are presented in Table 1 and hemodynamic quantities are presented in Table 2. More specific demographic information is provided in Table 3. Among these donors, two eyes from one donor (Cases 6 and 7) were diagnosed with neovascular AMD antemortem. The posterior punches from these eyes were acquired outside of the macula and appeared histopathologically normal.

**Table 1:** Anatomic parameters of case eyes.

Case	Age (years)	Area (mm$^2$)	CC density (%)	Ave CC diameter (µm)	A density (#/mm$^2$)	V density (#/mm$^2$)
1	88	5.0	74.4	17.2	15.7	30.1
2	67	3.4	73.1	17.7	9.9	23.4
3	88	1.2	71.6	19.1	7.4	12.3
4	$>89$	2.9	71.8	19.7	7.1	27.2
5	88	1.3	64.9	18.1	6.2	11.6
6	75	5.0	73.0	21.0	5.2	12.6
7	75	4.9	68.1	20.5	6.2	14.0
8	$> 89$	4.4	74.5	19.0	3.6	11.9

*Area* area of the tissue studied, *CC density* density of choriocapillaris when viewed en-face and flattened in a 2-D plane, *Ave CC diameter* the average diameter of choriocapillaris. *A density* the number of arteriole junctions per mm$^2$ tissue, *V density* the number of venule junctions per mm$^2$ tissue.

**Table 2:** Hemodynamic variables of 8 donor eye cases.

Case	ESS (Pa)			Vel (mm/s)			P$^{Art}$-P (mmHg)		
	Ave	Mean	Med	Ave	Mean	Med	Ave	Mean	Med
1	1.84	0.91	0.73	1.33	0.61	0.71	6.5	6.5	6.7
2	1.78	0.68	0.41	1.44	0.35	0.37	7	7	7.4
3	1.79	0.73	0.48	0.97	0.45	0.54	7.2	7.2	7.5
4	3.71	2.06	1.31	1.70	0.69	0.89	8.2	8.2	8.7
5	1.26	0.52	0.33	2.00	0.80	0.97	7.5	7.5	7.9
6	1.40	0.55	0.42	1.51	0.61	0.73	7.4	7.4	7.9
7	1.23	0.47	0.38	2.48	1.29	1.50	7.9	7.9	7.8
8	1.06	0.38	0.30	2.00	0.77	0.91	7.4	7.4	8.3
Weighted average	1.69	0.75	0.54	1.73	0.73	0.86	7.4	7.4	7.7

*ESS* endothelial shear stress, *Vel* velocity, *P*$^{Art}$ pressure at arteriole inlet, *Ave* arithmetic mean, *Mean* geometric mean, *Med* median. Weighted average based on the analyzed area of each specimen.

Across all inner choroidal tissues analysed, the weighted average blood velocity was 1.73 mm/s. The weighted average ESS based on tissue area was 1.69 Pa, within the physiologic range of ESS findings in other microvascular beds^43–45^.

**Figure 2:**
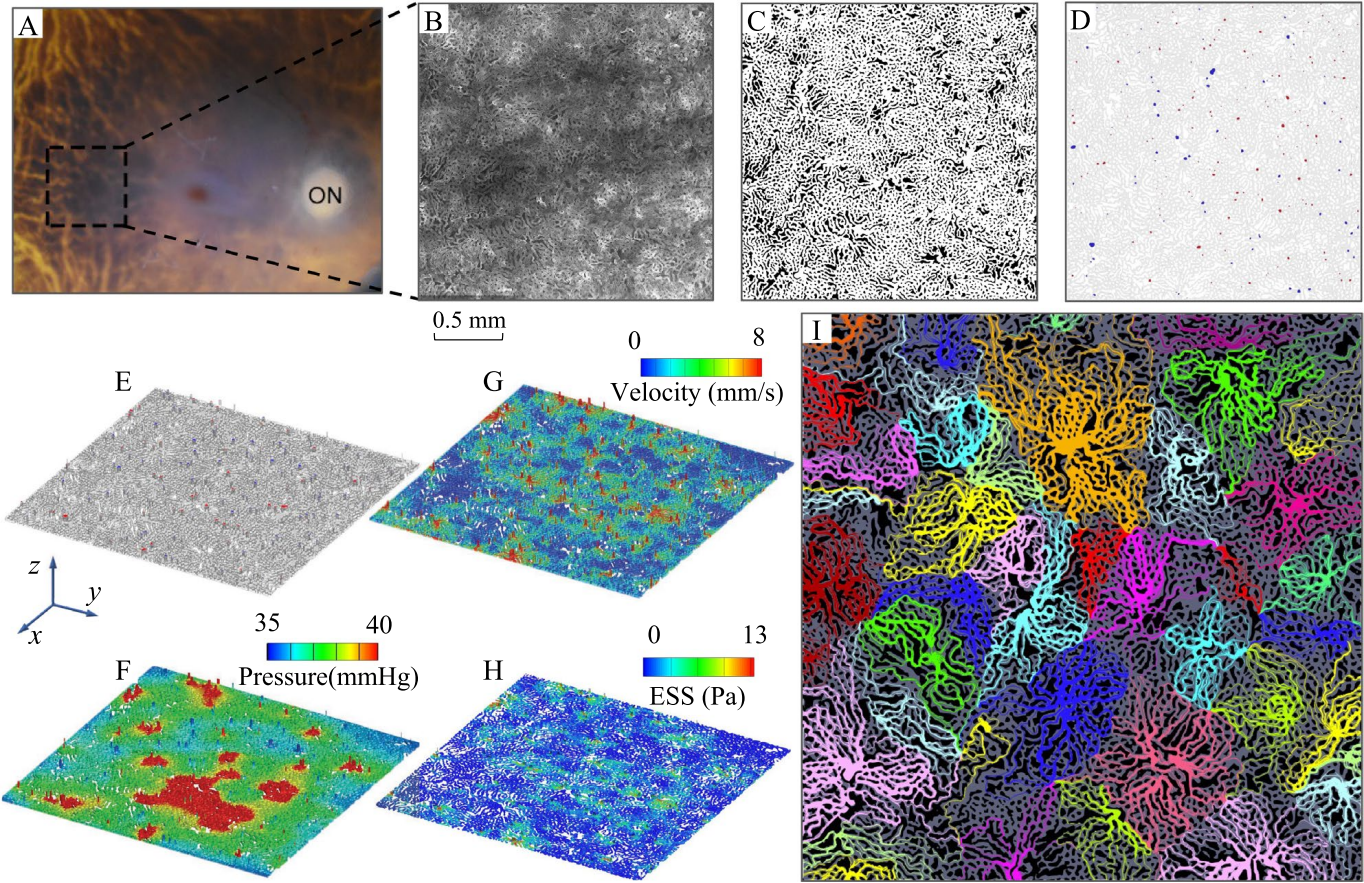
Illustration of ICH for Case #1 from donor image to 3-D hemodynamic fields. (**A**) Postmortem fundus image after removal of the anterior hemisphere. *ON* optic nerve. The dashed line indicates the approximate area from which the inner choroid was dissected. (**B**) En-face view of the 5.04 mm$^2$ area of choriocapillaris imaged by confocal microscopy after UEA lectin staining. This image was used to perform binary segmentation shown in (**C**), with vessels denoted by white and intercapillary pillars denoted by black. (**D**) Map of the junctions of Sattler’s layer arteriole (red) and venule (blue) junctions with the choriocapillaris. (**E**) 3-D reconstruction of the inner choroid. The view is from the posterior aspect to display the arteriole and venules. (**F**–**H**) Pressure, velocity, and ESS fields obtained from the InVascular simulation. (**I**) Lobular flow pattern in Case #1. Flow simulation of Case #1 in which streamlines originating from each arteriole are assigned a unique color.

As a representative case (Case #1 in Table 1), the right eye of an 88-year-old female donor was enucleated and fixed within 8 hours of death. Ocular history and gross examination revealed no indication of retinal disease (Fig. 2A). An approximately 5 mm$^2$ area from the posterior pole was labelled with Ulex Europaeus Agglutinin (UEA) lectin to visualize viable vascular structures, imaged in 3-D by fluorescent confocal microscopy (Fig. 2B), and segmented (Fig. 2C). The capillary density, measured as the percent area occupied by vessel lumens from an en face view was 74.4%, and the average capillary diameter was 17.2 µm. Images from deeper layers of Sattler’s layer arterioles and venules were used to manually identify a total of 79 arteriole junctions and 152 venule junctions (Fig. 2D). The capillary and arteriole/venule maps were used to construct the 3-D choriocapillary structure (blood flow domain) (Fig. 2E). Microscopy images and segmented capillary and arteriolar-venular anatomies are provided in (Fig. 9). This structure was then analysed by InVascular using the boundary conditions described above. The pressure distribution in the innermost plane (furthest from arterioles and venules), mid-layer velocity magnitude, and ESS are depicted in Fig. 2F–H. In the choriocapillaris, the average blood velocity was 1.3 mm/s. The pressure, velocity, and ESS fields displayed considerable spatial variability. Peak blood velocities were observed around the arteriole inlets. Blood entering arterioles dispersed radially while rapidly decelerating, followed by an acceleration corresponding to venular collection. ESS followed a similar distribution, with maxima centered on arteriole junctions, minima at watershed zones distant from arteriole and venule junctions, and local maxima of a lower magnitude at venule junctions.

We sought to assess the presence of lobules or vascular segments, which have been observed in the choroid of monkeys using fluorescent dyes^27,28^, and in the simplified model developed by Ref.^25^ (Fig. 1A). Like prior anatomic studies,^29^ we did not observe obvious evidence of functional lobules based on choroidal staining. Zouache et al.^25^, reconciled these findings by identifying highly regular separation lines arising from vascular units comprised of a central arteriole surrounded by more or less regularly spaced venules. To assess whether functional lobules could also be observed in realistic human-derived inner choroidal anatomy, we performed a simulation in which each arteriole was fed with a unique synthetic *dye*, and monitored the extent to which individual capillaries carried particles originating from different arterioles (Fig. 2I). As predicted from prior functional studies and models^20,23,25,28,29^, blood flow within individual capillaries was largely supplied by a single feeding arteriole, and the capillary bed formed networks of functional lobules. These findings confirm the presence of functional lobular units in the anatomically accurate human inner choroid.

### Influence of choriocapillary structure on hemodynamic quantities

**Figure 3:**
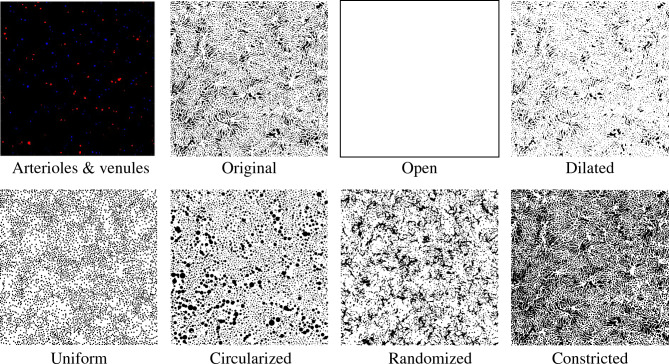
In the first panel, red and blue dots represent the arteriole inlets and venous outlets respectively (where these larger vessels connect to the capillary layer) relative to the capillary plane. These were held constant while adjusting the capillary arrangement as shown in the binary representations of capillary permutations with distinct geometries and capillary densities.

The choriocapillaris structure is dynamic with age and in disease settings. For example, histopathological findings suggest that capillary density is reduced in age and disease conditions^8,46–51^. Therefore, we evaluated the effects of choriocapillary geometry and density on the hemodynamics of the inner choroid. Using the representative case described in detail above, we performed six spatial permutations on the choriocapillaris structure, keeping the arteriole and venule distribution constant (Fig. 3). They are described as follows:

- *Dilated*: reduced the size of the intercapillary pillars radially by 2.5 microns, thereby increasing capillary diameters
- *Constricted*: enlarged the intercapillary pillars radially by 2.5 microns, effectively constricting the capillaries
- *Circularized*: changed the shape of the pillars by individually replacing each pillar with a circle of an equivalent area
- *Uniform*: replaced each intercapillary pillar with a circle of a uniform area corresponding to the average area of all intercapillary pillars
- *Random*: kept the intercapillary pillar size and shape constant, but repositioned them randomly throughout the capillary plane
- *Open*: eliminated the avascular intercapillary pillars entirely modelling the capillary layer as an empty, open plane

**Figure 4:**
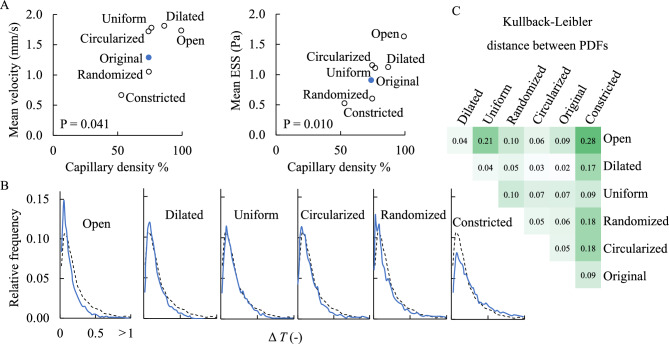
Capillary permutations on hemodynamic variables in Case #1. (**A**) Mean velocity (top) and Mean ESS (bottom) plotted against capillary density for each capillary permutation. (**B**) PDFs for each capillary permutation. The dashed line corresponds to the original PDF for Case #1. (**C**) Pairwise Kullback-Leibler distances for each capillary permutation.

The capillary density ranged from 53% (Constricted) to 100% (Open). We assessed the relationship between capillary density and hemodynamic variables, finding a significant positive relationship between capillary density and both the mean velocity and the mean ESS (Fig. 4A). We calculated the PDF for the transit time from arteriole to venule for > 2700 observations for each permutation, finding that the capillary structure significantly affected the distribution of particle transit times (Fig. 4B). At the extremes of capillary density, the Open configuration reduced the duration of particle transit and the Constricted configuration increased transit times compared to the original patient-derived geometry. However, the PDF was less sensitive to other intercapillary shapes, as estimated by the Kullback-Leibler distances, a measure of the relatedness of two probability density distributions (Fig. 4C).

**Figure 5:**
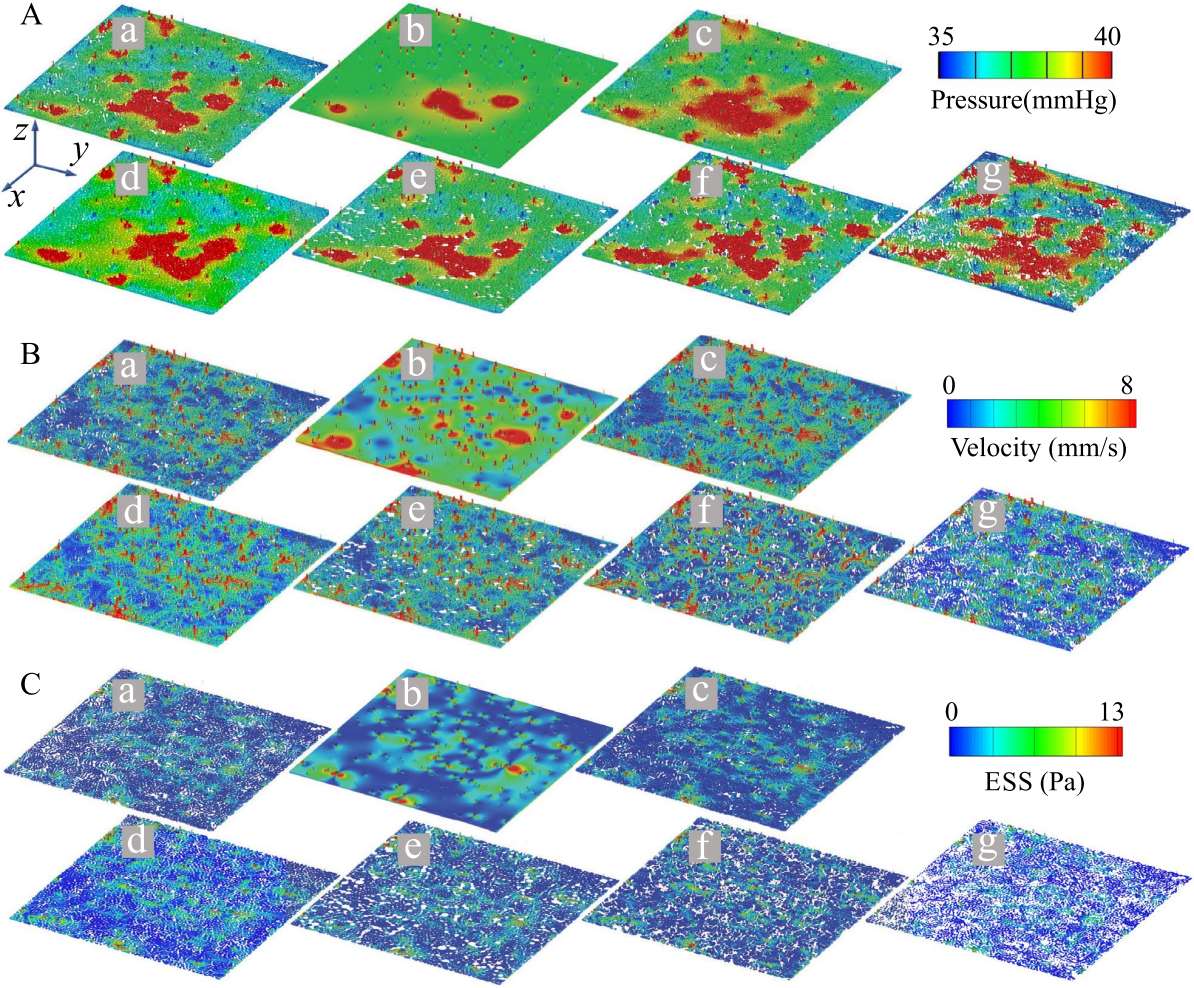
Fields of choriocapillary permutations in Case #1. (**A**) Pressure, (**B**) Velocity magnitude, and (**C**) ESS. (a) Original, (b) Open, (c) Dilated, (d) Uniform, (e) Circularized, (f) Randomized, (g) Constricted.

Qualitatively, the distributions of pressure, velocity, and ESS followed a similar pattern irrespective of capillary structure, with local maxima centered on arteriole and venule junctions (Fig. 5). In the Open configuration, the velocity and ESS were more uniform, with fewer and smaller areas of low velocity (< 1 mm/s) and ESS (< 4 Pa). Conversely, the Constricted configuration exhibited more abrupt reductions in velocity and ESS proximal to the arteriole inlets, resulting in a less homogenous flow pattern, and greater areas of low velocity and ESS.

### Influence of arteriole/venule distribution on hemodynamic variables

**Figure 6:**
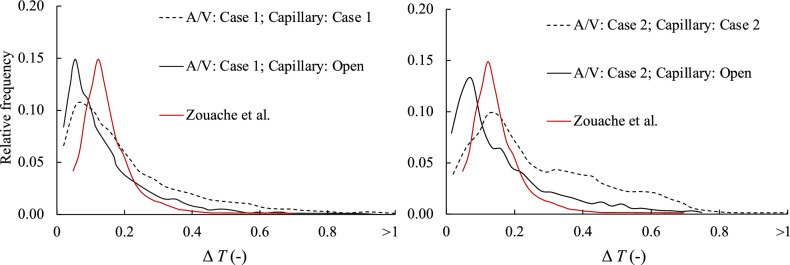
Influence of arteriole/venular arrangement on fluid-particle transit in the choriocapillaris. PDF for three geometries. The black dashed line corresponds to the original capillary and arteriole/venule maps for Cases #1 and #2. The black solid line corresponds to an open capillary arrangement with the arteriole/venule map from Cases #1 and #2. The red solid line corresponds to the Zouache et al., geometry: an open capillary layer combined with a quasirandom arteriole and venule arrangement.

Finally, we sought to assess the effects of irregular arteriolar/venular spacing on inner choroidal hemodynamics. We compared the designed arteriolar/venular distribution developed by Zouache et al.^25^ with a patient-derived distribution described above, using the open capillary configuration in both. Anatomically-derived arteriole/venule distribution led to significantly more heterogeneous transit time PDF (Fig. 6). In this anatomically-derived arrangement, we observed a higher frequency of relatively short (< 0.1) and long (> 0.3) transit times. This likely owes to the observation that in human inner choroidal anatomies, oftentimes arteriole and venular junctions are quite close together, leading to a high frequency of very short intercapillary transits. Similarly, the irregular spacing in human anatomies also leads to increased transit times for blood entering arterioles that are surrounded by other arterioles and thus experiencing low-pressure gradients, or that are distant from venules. The Kullback-Leibler distance between the PDFs of the idealized and human-derived arteriolar/venular arrangement was 0.22, which was similar to the distance between the most extreme capillary permutations, holding the arteriole/venule arrangement constant. To assess whether this was unique to the arteriole/venular arrangements in Case #1, we repeated this analysis for Case #2, and observed a similar pattern (Fig. 6). This suggests that for the permutations studied, both the intercapillary and arteriolar/venular arrangements play an approximately equal role in determining the PDF of an inner choroidal vascular bed.

## Discussion

We have demonstrated the reliability of InVascular for revealing the hemodynamic characteristics in designed and real human choriocapillaris. The flow pattern and PDFs of the travel time of a corpuscle in a functional vascular segment of choriocapillaris were in good quantitative agreement with those from Zouache’s outcomes. Meanwhile, the weighted velocities over 8 donor cases agreed with human measurements, and ESS values were within the reported physiologic range for other microvascular beds^43^.

Our principal finding is that donor-specific arteriolar and venular distribution and intercapillary pillar structures are equally important in affecting the distribution of blood flow within individual eyes. In diseases of ageing such as AMD, choriocapillary density and individual capillary diameters are reduced^8,46,48–51^. Our model suggests that such changes could influence the distribution of blood flow both by reducing the overall blood velocity within choriocapillaris, and by increasing its spatial heterogeneity. As blood velocity decreases, so too does the transport of oxygen, nutrients, and metabolic waste. These findings support a model in which capillary constriction or involution results in the failure of the inner choroid to fulfil its transport demand, and thereby contribute to the development of retinal pathologies. Our findings also suggest that these transport functions may be most sensitive to capillary constrictions for those capillaries relatively distant from arteriolar/venular junctions, whose velocity is already significantly lower than those close to these junctions.

Histopathologic evidence also suggests that Sattler’s layer feeding arterioles can undergo atherosclerotic and hypertensive changes, which are associated with AMD^8,46^. To the extent that these changes affect the blood transport function of those feeding arterioles, our findings support the concept that such changes could have profound influences on inner choroidal blood flow distribution. In recent years, choroidal hemodynamic imaging in patients has advanced dramatically, enabling clinical researchers to assess the relationship between inner choroidal perfusion and the progression of diseases such as AMD. The analysis presented here complements these efforts by providing insights into how anatomic factors dictate local choroidal hemodynamics. In addition, future studies may clarify the extent to which inner choroid hemodynamics support retinal health and disease.

Our model revealed obvious functional lobules or vascular segments, which have been observed in the eyes of monkeys using fluorescent dyes^27,28^, but whose nature has remained controversial because of the lack of anatomic evidence for such independent units in humans^29^. In agreement with prior anatomic studies, we did not observe regularity in the arteriolar/venular distribution. Nonetheless, the resulting flow dynamics resulted in the development of functional vascular units such as those observed in dye-based monkey studies, providing support for the model presented by Zouache et al.^25^ in which functional vascular units can exist without physical barriers by pressure-driven separation lines preventing communication between units.

Overall strengths of the study include the use of high-quality human donor eye specimens, the extraction of human anatomically-derived geometries, and the implementation of the VLBM as a flexible, computationally efficient, and accurate fluid dynamics modelling system. Our study also has several limitations. Among these are simplifying assumptions such as the rigid, flat tissue structure, constant and equivalent pressure gradients between arteriolar inlet and venular outlets, arteriolar and venular junctions being orthogonal to the capillary plane, and the modelling of blood as a homogenous Newtonian fluid. We also did not account for blood pulsatility, which is observed and heterogeneous in human choroidal veins^52^. In addition, because substantial regional heterogeneity exists in choroidal anatomy^53^, and our study focused on posterior pole, our findings may not be generalizable to inner choroidal hemodynamics in the peripheral, macular, or foveal regions. Using cadaver eyes is also a limitation in that death and fixation may affect the anatomic structure. Consequently, the present models may be best viewed as an approximation of instantaneous choroidal blood flow, which is a dynamic process. Another limitation of this study is the exclusive use of aged donors. Choroidal density and capillary diameter have been found to decline with age^51^. Consequently, the extent to which our findings apply to choroidal anatomies in younger eyes may be the subject of a separate analysis. Despite these limitations, this methodology improves upon previous efforts by implementing more accurate models of human inner choroidal anatomy.

## Methods

### Donor eyes

All studies on human tissue followed the guidelines of the Declaration of Helsinki. The study of deidentified tissue from deceased individuals obtained from various eye banks in the United States, who received permissions to collect cadaver tissues. The study was exempted from IRB review by the University of Virginia Institutional Review Board for Health Sciences Research in accordance with the U.S. Health and Human Services human subjects regulations. Eyes were enucleated with a death-to-preservation time of less than or equal to 12 h. A small incision in the limbus was made to permit media exchange. Eyes were fixed in 10% buffered formalin at 4 $^\circ$C for 24–48 h, then transferred to 70% ethanol and stored at 4 $^\circ$C until use.

**Table 3:** The information regarding the donor and the circumstances leading to their passing.

Case ID	Sex	Race	Cause of death
1	F	Caucasian	Cerebral vascular accident
2	M	Caucasian	Metastatic lung cancer
3	F	Caucasian	Cerebral vascular accident
4	F	Caucasian	Chronic obstructive pulmonary disease
5	F	Caucasian	Pneumonia
6	F	Caucasian	Respiratory failure
7	F	Caucasian	Respiratory failure
8	F	Caucasian	Myocardial infarction

### Overview of InVascular from image acquisition to hemodynamic distributionsin donor-specific human choroid

**Figure 7:**
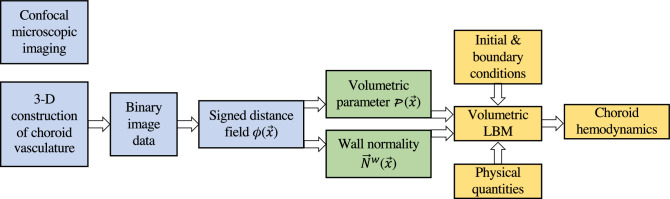
Flowchart from confocal microscopic imaging to choroid hemodynamics. The volumetric parameter and wall normality (green) are the outputs of image processing (blue) and inputs of CFD using VLBM (yellow), enabling seamless connection between both.

As schematized in Fig. 7, InVascular, from confocal microscopic imaging of a human eye donor to chroid hemodynamics, consists of three components: (1) image acquisition and 3-D construction (blue), (2) determination of volumetric parameter and wall normality (green), and (3) VLBM together with physical and computational conditions for ESS (yellow). The unique feature of InVascular is that component (2) is the output of component (1) and the input of component (3), enabling seamless connection between the components (1) and (3). InVascular has been our continuous efforts from its original kinetic-based VLBM^30,32^, imaged-based computational fluid dynamics^31,32^, to ICH for medical applications[a,b], together with GPU parallel computing^54,55^. In this section, we present the first component. Components (2) and (3) are referred to reference^32^ from the same group.

The process from confocal microscopic imaging of a human eye donor to ESS distribution on the inner wall of choriocapillaris consists of three components: image acquisition and 3-D construction (blue), determination of volumetric parameter, and wall normality (green), and VLBM together with physical and computational conditions for ESS (yellow). The first two components are seamlessly connected as the outputs of the image process, i.e., the volumetric parameter and wall normality, are the inputs of the VLBM. In this section, we present the modeling and formulation from three aspects: image acquisition and processing, formulation of volumetric lattice Boltzmann equations (VLBEs), and en-route calculation of ESS. It is noted that the streaming operation in the VLBEs is generalized from its original formula^30^.

**Figure 8:**
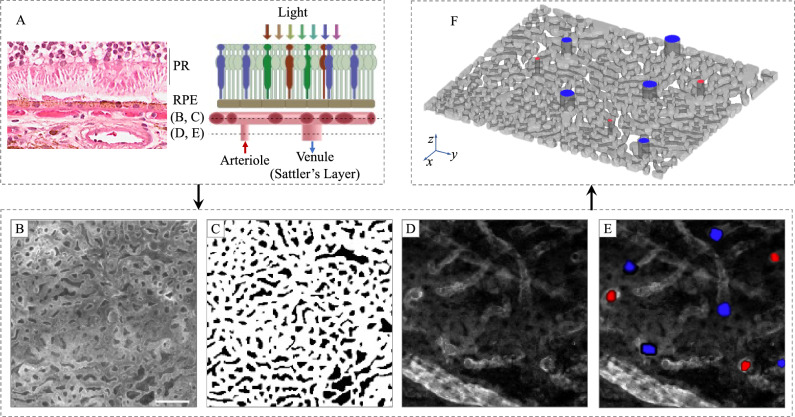
Segmentation of human inner choroid. (**A**) Hematoxylin and eosin-stained cross-section of a human eye (left) and schematic of photoreceptor/RPE/choroid complex (right). Light is focused on the photoreceptor (PR) layer, which rests on a monolayer of retinal pigmented epithelial cells (RPE). Posterior to the RPE is Bruchs membrane, which separates the RPE from the the inner choroid. The inner choroid is comprised of a plane of capillaries, choriocapillaris, which is fed and drained by arterioles and venules of Sattler’s layer. Dashed lines correspond to the plane of images in subsequent panels. (**B**) Confocal fluorescent micrograph of a human donor eye at the depth of choriocapillaris. (**C**) Results of binary segmentation of (**B**), with capillaries labeled 1 (white) and intercapillary pillars labeled 0 (black). (**D**) Confocal fluorescent micrograph at a depth showing arteriolar and venular junctions with choriocapillaris, visible as brightly outlined hollow voids. This image was acquired from the same location as (**B**). (**E**) Result of segmentation of arterioles (red) and venules (blue) from (**D**). (**F**) 3-D reconstruction of the inner choroid from a human donor eye viewed from the posterior aspect. Scale bar 100 µm.

### Image acquisition of choroidal vasculature

The anterior chamber was removed, and a biopsy punch (6–10 mm diameter depending on the tissue) was used to excise the sclera, choroid, RPE, and retinal layers. The RPE/choroid was isolated and the autofluorescent RPE monolayer was mechanically debrided using Weck-Cel surgical sponges. Choroid tissue, including Bruch’s membrane, was separated from the sclera, hydrated, then blocked in a solution containing 2% normal donkey serum (Jackson Immuno), 1%BSA (stabilizer), 0.1% Triton X-100 (penetration enhancer), 0.05% Tween 20 (detergent and surface tension reducer), 0.05% sodium azide (preservative) diluted in 1X PBS (w/o Ca$^{2+}$/Mg$^{2+}$), pH 7.2. The tissue was blocked for approximately 4 hours at room temperature with gentle agitation. Then, tissue was transferred to a clean tube containing FITC-conjugated UEA lectin (Sigma, cat# L9006) diluted 1:50 in fresh blocking solution. The tissue was incubated overnight at 4 $^\circ$C in the dark with gentle agitation. The tissue was washed with three exchanges of PBS-T (1X PBS + 0.1% Tween 20) for a total of 1 h. The tissue was then washed with two exchanges of 1 $\times$ PBS and one exchange of deionized water. Tissue was mounted to a slide using ProLong Gold Antifade Mountant (Invitrogen), with choriocapillaris oriented towards the coverslip. UEA lectin-labeled choroidal vessels were imaged by confocal microscopy (Nikon A1R confocal microscope) using a 10 $\times$ objective. Z-stacks were acquired and the entire thickness of choroidal tissue was imaged. The Z plane resolution ranged from 0.7 to 3.1 micron. The range of images acquired in Z ranged from 29.4 to 93 micron. It should be noted that the range depended strongly on the “flatness” of the tissue, and should not be considered as a proxy for anatomic thickness.

### Image processing for a 3-D choroid vasculature with arterioles/venules as inlets/outlets

The anatomic reconstruction of the 3-D choroid vasculature is performed in the following using MATLAB programming, starting from the acquired image for the inner choroid layer. Manual involvement has been minimized in this process. Note that the following represents the optimized segmentation method. Earlier iterations of these methods involving manual segmentation of microscope images were used to generate some of the vascular networks whose simulations are reported.

**Figure 9:**
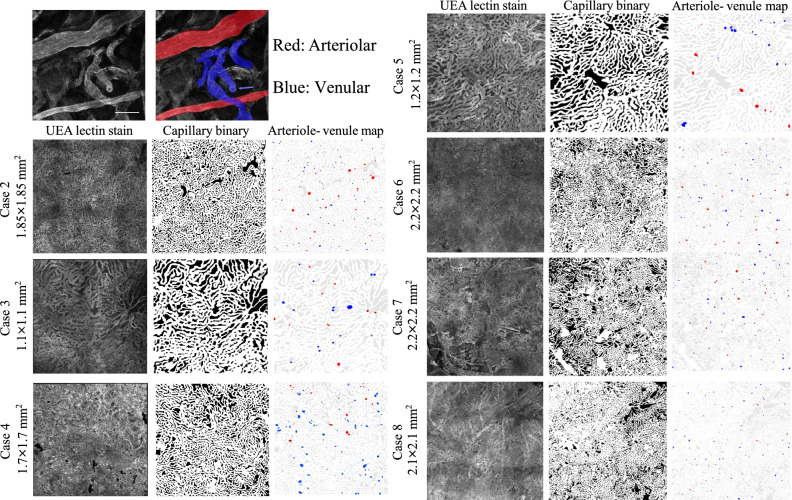
Arteriole and venule identification was performed manually. The left panel shows a single Z slice acquired just posterior to the capillary plane with arterioles and venules present. The right panel denotes arteriolar (red) and venular (blue) identities. Note the arterioles are defined by elongated endothelial shape, narrow diameter, and relatively straight vessel shape. In contrast, venules are widened, tortuous, and have wider endothelial borders. The blue arrow denotes a venular-capillary junction (capillary plane is not visible, but anterior to this plane). Arteriolar and venular identities were identified with 75–87% consistency between two masked graders.

- **Anatomically extracting the choriocapillary structure**. A histologic and schematic representation the region of interest is presented in (Fig. 8A). The region of interest comprising approximately 2.5 mm $\times$ 2.5 mm of the inner choroid is projected to a flat plane. The images comprising the capillary layer (Fig. 8B) are compressed into a single X–Y projection in an 8-bit grayscale image. The grayscale image is further converted into a binary image (Fig. 8C) by assigning the capillary structure pixels as 0 and capillary void pixels as 1. This process was achieved in a semi-automated fashion and had a high degree of interoperator consistency with 85–90% pixel identity among three independent blinded operators.
- **Locating arterioles and venules on choriocapillaris**. Arteriole and venule junctions into the capillary layer were manually identified and segmented from images of the deeper choroidal layers, immediately posterior to the capillary layer (Fig. 8D,E). Arteriolar/venular identity was assigned based on vessel diameter, the shape/orientation of the endothelium, and the vessel tortuosity. An example is illustrated in Fig. 9.
- **Generating a series of eroded image layers to form the capillary layer**. Setting the choriocapillary layer in 1) as the middle of the vasculature, we symmetrically erode the capillary layer in the opposite directions using function strel (*disk*, r, n), which creates a disk-shaped structuring element with r the radius and n the number of line structuring elements. The eroding radius is set *round* to mimic the tube shape of a capillary. After the erosion operation, a series of image layers are generated. The number of the eroded layers is determined by the thickness of the choroid, i.e., 10 µm.
- **Forming a 3-D choroid vasculature with arteriole inlets and venule outlets**. The series of eroded image layers are stacked in the vertical direction and the top and bottom capillary voids are closed by adding two layers with all intercapillary structures, respectively. The arterioles and venules are mounted on the top layer of the image stack and the pixels of arterioles and venules are reopened to allow inflow and outflow of the blood. The 3-D network of choriocapillaris together with arterioles and venules is completed by extending the basal-most layer to approximately 35 µm, as shown in Fig. 8F.

### Formulation of VLBEs

In the VLBM, fluid particles are uniformly distributed in lattice cells instead of sitting at lattice grids as in conventional LBM. Each cell is labeled by its occupation of solid volume, referred to volumetric parameter ${\mathcal {P}}(\varvec{x},t)\left( \equiv V_{solid}(\varvec{x},t)/V_{total}(\varvec{x},t)\right)$. The value of ${\mathcal {P}}$ distinguishes three types of lattice cells in the simualtion domian. They are solid cell (${\mathcal {P}}$ = 1), fluid cell (${\mathcal {P}}$ = 0), and boundary cell (0 > ${\mathcal {P}}$ > 1). The formulation of the VLBM is self-regularized to deal with the complex flow geometries through ${\mathcal {P}}$. The VLBM deals with the time evolution of the particle population $n_i(\varvec{x},t)$ with discrete velocity $\varvec{e}_i$ along the *i*th direction in each lattice cell. The volumetric lattice Boltzmann equation with BGK (Bhatnagar, Gross, and Krook) approximation for the collision operation reads:

1 $$\begin{aligned} n_i\left( \varvec{x}+\varvec{e}_i{\delta t},t\right) -n_i\left( \varvec{x},t\right) =-\frac{1}{\tau }\left[ n_i\left( \varvec{x},t\right) -n_i^{{eq}}\left( \varvec{x}, t\right) \right] +F_i\left( \varvec{x},t\right) , \ \ \ \ i=0 - b, \end{aligned}$$

where $n_i\left( \varvec{x},t\right)$, $n_i^{{eq}}\left( \varvec{x},t\right)$, $\tau$, and $F_i\left( \varvec{x},t\right)$ are the particle population, the equilibrium particle population, the relaxation time due to fluid-particle collisions, and the discrete forcing term, respectively, in cell $\varvec{x}$ at time *t* and *b* is the number of discrete molecular velocity determined by the selected lattice model. The forcing term is formulated as:

2 $$\begin{aligned} F_i\left( \varvec{x},t\right) =-\omega _iN\frac{\varvec{e}_i\cdot \varvec{a}\left( \varvec{x},t\right) }{c_s^2}{\delta t}. \end{aligned}$$

The equilibria for incompressible flows are:

3 $$\begin{aligned} n_i^{{eq}}\left( \varvec{x},t\right) ={N\omega }_i\left\{ 1+\frac{\varvec{e}_i\cdot \varvec{u}}{c_s^2}+\frac{(\varvec{e}_i\cdot \varvec{u})^2}{2c_s^4}-\frac{\varvec{u}\cdot \varvec{u}}{2c_s^2}\right\} , \end{aligned}$$

where $\varvec{a}$ is the acceleration due to an external force, $c_s$ and $\omega _i$ are the sound speed and weighting factor determined also by selected lattice model, and $N\left( \varvec{x},t\right) ({\equiv }\sum _{i=0}^bn_i\left( \varvec{x},t\right) {\equiv }\sum _{i=0}^bn_i^{{eq}}\left( \varvec{x},t\right) )$ and $\varvec{u}\left( \varvec{x},t\right) ({\equiv }\sum _{i=0}^b\varvec{e}_in_i\left( \varvec{x},t\right) /N\left( \varvec{x},t\right) {\equiv }\sum _{i=0}^b\varvec{e}_in_i^{{eq}}\left( \varvec{x},t\right) /N\left( \varvec{x},t\right) )$ are the zeroth and 1^st^ order moment of $n_i\left( \varvec{x},t\right)$, respectively. It is noted that the relation between the particle density distribution function $f_i\left( \varvec{x},t\right)$ and the particle population $n_i\left( \varvec{x},t\right)$ used in the node-based LBM and the cell-based VLBM, respectively, is $n_i\left( \varvec{x},t\right) =[1-{\mathcal {P}}\left( \varvec{x}\right) ]f_i(\varvec{x},t)$. Since $\Delta V_f\left( \varvec{x},t\right) =\left[ 1-{\mathcal {P}}\left( \varvec{x}\right) \right] \Delta V$, $n_i\left( \varvec{x},t\right)$ collapses to $f_i\left( \varvec{x},t\right)$ in fluid cells, where ${\mathcal {P}}=0$. In general, Eq. (1) consists of three operations: collision including the momentum exchange between the moving boundary and the flow, streaming accompanying a volumetric bounce-back procedure in boundary cells; and migrating volumetrically moving the residual fluid particles into the flow domain when the boundary swipes over a boundary cell toward a solid cell. In this work, we consider rigid capillary walls, thus Eq. (1) only involves the 1$^{st}$ two operators as follows.

Collision:

4 $$\begin{aligned} n'_i\left( \varvec{x},t\right) =n_i\left( \varvec{x},t\right) -\frac{1}{\tau }\left[ n_i\left( \varvec{x},t\right) -n_i^{{eq}}\left( \varvec{x},t\right) \right] +F_i\left( \varvec{x},t\right) , \end{aligned}$$

Streaming:

5 $$\begin{aligned} n_i\left( \varvec{x},t+{\delta t}\right) =G_i\left( \varvec{x}\right) \frac{1-{\mathcal {P}}\left( \varvec{x}\right) }{1-{\mathcal {P}}\left( \varvec{x}+\varvec{e}_{i*}{\delta t}\right) }n'_i\left( \varvec{x}+\varvec{e}_{i*}{\delta t},t\right) + \left[ 1+G_i\left( \varvec{x}\right) \right] \left[ n'_i\left( \varvec{x}+\varvec{e}_{i*}{\delta t},t\right) +\frac{{\mathcal {P}}\left( \varvec{x}+\varvec{e}_{i*}{\delta t}\right) -{\mathcal {P}}\left( \varvec{x}\right) }{1-{\mathcal {P}}\left( \varvec{x}\right) } n'_{i*}\left( \varvec{x},t\right) \right] , \end{aligned}$$

with a local structure parameter:

6 $$\begin{aligned} G_i\left( \varvec{x}\right) = \left\{ \begin{array}{lr} \text {1,}\ \ \ \ \text {if}\ \ {\mathcal {P}}\left( \varvec{x}+\varvec{e}_{i*}{\delta t}\right) < {\mathcal {P}}\left( \varvec{x}\right) &{} \\ \text {0,}\ \ \ \ \text {if} \ \ {\mathcal {P}}\left( \varvec{x}+\varvec{e}_{i*}{\delta t}\right) \ge {\mathcal {P}}\left( \varvec{x}\right) . \end{array} \right. \end{aligned}$$

It is noted that Eq. (5) has contain the bounce-back (no slip) boundary condition in the boundary cells, a detail explanation of which can be found in published papers. The resulting density, velocity, and pressure of the flow system are obtained as follows:

7 $$\begin{aligned} \rho \left( \varvec{x},t\right){} & {} =\frac{\sum _{i=0}^bn_i\left( \varvec{x},t\right) }{1-{\mathcal {P}}(\varvec{x})}, \end{aligned}$$

8 $$\begin{aligned} \varvec{u}\left( \varvec{x},t\right){} & {} =\frac{\sum _{i=0}^b\varvec{e}_in_i\left( \varvec{x},t\right) }{\sum _{i=0}^bn_i\left( \varvec{x},t\right) }, \end{aligned}$$

9 $$\begin{aligned} p\left( \varvec{x},t\right) -p_0{} & {} =c_s^2[\rho \left( \varvec{x},t\right) -\rho _0], \end{aligned}$$

where $p_0$ and $\rho _0$ (=1) are reference pressure and density in lattice units, respectively.

### En-route calculation of ESS

In hydrodynamics, the strain-rate tensor is defined as:

10 $$\begin{aligned} S_{\alpha \beta }=\frac{1}{2}\left( \frac{{\partial }u_{\alpha }}{{\partial }x_{\beta }}+\frac{{\partial }u_{\beta }}{{\partial }x_{\alpha }}\right) ,\ \ \alpha ,\beta =1,2,3. \end{aligned}$$

It is calculated in the postprocessing using finite difference method after the velocity field $\varvec{u}\left( \varvec{x},t\right)$ is computed. In the VLBM, $S_{{\alpha \beta }}$ can also be obtained from the 2^nd^ order moment of the particle populations as follows:

11 $$\begin{aligned} S_{\alpha \beta }=\frac{-1}{2\tau c_s^2\sum _{i=0}^bn_i\left( \varvec{x},t\right) }\sum _{i=0}^b\varvec{e}_{{i\alpha }}\varvec{e}_{{i\beta }}\left( n_i-n_i^{{eq}}\right) . \end{aligned}$$

It has been demonstrated that Eq. (11) is more robust than Eq. (10) to calculate $S_{\alpha \beta }$.

The total stress tensor is formulated as:

12 $$\begin{aligned} T_{\alpha \beta }=\frac{-\sum _{i=0}^bn_i\left( \varvec{ x},t\right) c_s^2}{1-{\mathcal {P}}}\delta _{{\alpha \beta }}+\left( 2\tau -1\right) c_s^2S_{{\alpha \beta }}, \end{aligned}$$

where $\delta _{{\alpha \beta }}$ is the Kronecker unit tensor. The Cauchy formula gives the overall stress on the wall with a normal vector $\varvec{N}^w$:

13 $$\begin{aligned} T_{\alpha }^{(W)}=T_{{\alpha \beta }}N_{\beta }^w, \end{aligned}$$

where Einstein summation convention with index notation has been used. Then its projection onto the normal direction ($\varvec{n}$) yields the endothelial normal stress (ENS):

14 $$\begin{aligned} T_{\alpha }^{({ENS})}=(N_{\beta }^wT_{\gamma \beta }N_{\gamma }^w)N_{\alpha }^w, \end{aligned}$$

where $\gamma \ (=1,2,3)$ is also an index for the axial direction in the 3-D Cartesian coordinate system.The ESS is then computed as the difference between the overall stress and its projection onto the normal:

15 $$\begin{aligned} T_{\alpha }^{({ESS})}=T_{{\alpha \beta }}N_{\beta }^w-(n_{\beta }T_{\gamma \beta }n_{\gamma })n_{\alpha }. \end{aligned}$$

With the determination of the local wall normality $\varvec{N}^w$, both ESS and ENS (when needed) can be obtained en-route during the VLBM implementation. A detailed presentation of this formulation can be found in a published paper^32^.

## Data Availability

Data and code that support the figures and conclusions presented in the manuscript will be provided upon reasonable request to the corresponding author.

## Abbreviations

AMD: Age-related macular degeneration
CC: Choriocapillaris
CFD: Computational fluid dynamics
ESS: Endothelial shear stress
GPU: Graphics Processing Unit
ICH: Image-based computational hemodynamics
ICGA: Indocyanine green angiography
LDF: Laser Doppler flowmetry
LBM: Lattice Boltzmann method
OCTA: Optical coherence tomography angiography
ON: Optic nerve
PR: Photoreceptor
PDF: Probability density function
RPE: Retinal pigmented epithelial
UEA: Ulex Europaeus Agglutinin
VLBE: Volumetric lattice Boltzmann equation
VLBM: Volumetric lattice Boltzmann method

## References

[1] Nickla DL, Wallman J (2010). The multifunctional choroid. Prog. Retin. Eye Res..

[2] Xu H (2002). Improved leukocyte tracking in mouse retinal and choroidal circulation. Exp. Eye Res..

[3] Omri S (2011). Microglia/macrophages migrate through retinal epithelium barrier by a transcellular route in diabetic retinopathy: role of PKC$$\zeta $$ in the goto kakizaki rat model. Am. J. Pathol..

[4] Penfold P, Killingsworth M, Sarks S (1985). Senile macular degeneration: the involvement of immunocompetent cells. Graefes Arch. Clin. Exp. Ophthalmol..

[5] Sakurai E, Anand A, Ambati BK, van Rooijen N, Ambati J (2003). Macrophage depletion inhibits experimental choroidal neovascularization. Investig. Ophthalmol. Vis. Sci..

[6] Sakurai E (2003). Targeted disruption of the CD18 or ICAM-1 gene inhibits choroidal neovascularization. Investig. Ophthalmol. Vis. Sci..

[7] Ogura S (2020). A role for mast cells in geographic atrophy. FASEB J..

[8] McLeod DS (2009). Relationship between rpe and choriocapillaris in age-related macular degeneration. Investig. Ophthalmol. Vis. Sci..

[9] Sohn EH (2019). Choriocapillaris degeneration in geographic atrophy. Am. J. Pathol..

[10] Seddon JM (2016). Histopathological insights into choroidal vascular loss in clinically documented cases of age-related macular degeneration. JAMA Ophthalmol..

[11] Biesemeier A, Taubitz T, Julien S, Yoeruek E, Schraermeyer U (2014). Choriocapillaris breakdown precedes retinal degeneration in age-related macular degeneration. Neurobiol. Aging.

[12] Metelitsina TI (2008). Foveolar choroidal circulation and choroidal neovascularization in age-related macular degeneration. Investig. Ophthalmol. Vis. Sci..

[13] Xu W (2010). Association of risk factors for choroidal neovascularization in age-related macular degeneration with decreased foveolar choroidal circulation. Am. J. Ophthalmol..

[14] Grunwald JE (1998). Foveolar choroidal blood flow in age-related macular degeneration. Investig. Ophthalmol. Vis. Sci..

[15] Nassisi M (2019). Choriocapillaris impairment around the atrophic lesions in patients with geographic atrophy: A swept-source optical coherence tomography angiography study. Br. J. Ophthalmol..

[16] Alagorie AR, Verma A, Nassisi M, Sadda SR (2019). Quantitative assessment of choriocapillaris flow deficits in eyes with advanced age-related macular degeneration versus healthy eyes. Am. J. Ophthalmol..

[17] Nassisi M, Baghdasaryan E, Borrelli E, Ip M, Sadda SR (2019). Choriocapillaris flow impairment surrounding geographic atrophy correlates with disease progression. PLoS ONE.

[18] Thulliez M (2019). Correlations between choriocapillaris flow deficits around geographic atrophy and enlargement rates based on swept-source OCT imaging. Ophthalmol. Retina.

[19] Nassisi M, Tepelus T, Nittala MG, Sadda SR (2019). Choriocapillaris flow impairment predicts the development and enlargement of drusen. Graefes Arch. Clin. Exp. Ophthalmol..

[20] Boltz A (2010). Choroidal blood flow and progression of age-related macular degeneration in the fellow eye in patients with unilateral choroidal neovascularization. Investig. Ophthalmol. Vis. Sci..

[21] Spaide RF (2016). Choriocapillaris flow features follow a power law distribution: implications for characterization and mechanisms of disease progression. Am. J. Ophthalmol..

[22] Hecht A (2020). Relationship between morphological and vascular alterations in geographic atrophy using a multimodal imaging approach. Acta Ophthalmol..

[23] Zouache M, Eames I, Luthert P (2015). Blood flow in the choriocapillaris. J. Fluid Mech..

[24] Flower R (2001). Theoretical investigation of the role of choriocapillaris blood flow in treatment of subfoveal choroidal neovascularization associated with age-related macular degeneration. Am. J. Ophthalmol..

[25] Zouache M, Eames I, Klettner C, Luthert P (2016). Form, shape and function: Segmented blood flow in the choriocapillaris. Sci. Rep..

[26] Lee JE (2017). Functional end-arterial circulation of the choroid assessed by using fat embolism and electric circuit simulation. Sci. Rep..

[27] Hayreh SS (1975). Segmental nature of the choroidal vasculature. Br. J. Ophthalmol..

[28] Hayreh SS (1974). Recent advances in fluorescein fundus angiography. Br. J. Ophthalmol..

[29] Edwards M, Lutty GA (2021). Bruch’s membrane and the choroid in age-related macular degeneration. Age Relat. Mac. Degen..

[30] Yu H (2014). Mass-conserved volumetric lattice Boltzmann method for complex flows with willfully moving boundaries. Phys. Rev. E.

[31] An S, Yu HW, Wang Z, Kapadia B, Yao J (2017). Unified mesoscopic modeling and GPU-accelerated computational method for image-based pore-scale porous media flows. Int. J. Heat Mass Transf..

[32] Zhang X (2022). Volumetric lattice Boltzmann method for wall stresses of image-based pulsatile flows. Sci. Rep..

[33] An S, Zhan Y, Yao J, Yu HW, Niasar V (2020). A greyscale volumetric lattice Boltzmann method for upscaling pore-scale two-phase flow. Adv. Water Resour..

[34] Chen R, Shao J-G, Zheng Y-Q, Yu H-D, Xu Y-S (2013). Lattice Boltzmann simulation for complex flow in a solar wall. Commun. Theor. Phys..

[35] Chen R, Yu H, Zhu L, Patil RM, Lee T (2017). Spatial and temporal scaling of unequal microbubble coalescence. AIChE J..

[36] Chen R, Yu HW, Zeng J, Zhu L (2020). General power-law temporal scaling for unequal-size microbubble coalescence. Phys. Rev. E.

[37] Yu H, Girimaji SS (2005). Near-field turbulent simulations of rectangular jets using lattice Boltzmann method. Phys. Fluids.

[38] Yu H, Girimaji SS, Luo L-S (2005). DNS and LES of decaying isotropic turbulence with and without frame rotation using lattice Boltzmann method. J. Comput. Phys..

[39] Gelfand BD, Ambati J (2016). A revised hemodynamic theory of age-related macular degeneration. Trends Mol. Med..

[40] Kiryu J (1994). Noninvasive visualization of the choriocapillaris and its dynamic filling. Investig. Ophthalmol. Vis. Sci..

[41] Takasu I, Shiraga F, Okanouchi T, Tsuchida Y, Ohtsuki H (2000). Evaluation of leukocyte dynamics in choroidal circulation with indocyanine green-stained leukocytes. Investig. Ophthalmol. Vis. Sci..

[42] Flower RW, Fryczkowski AW, McLeod DS (1995). Variability in choriocapillaris blood flow distribution. Investig. Ophthalmol. Vis. Sci..

[43] Santamaría R, González-Álvarez M, Delgado R, Esteban S, Arroyo AG (2020). Remodeling of the microvasculature: May the blood flow be with you. Front. Physiol..

[44] Bernabeu MO (2014). Computer simulations reveal complex distribution of haemodynamic forces in a mouse retina model of angiogenesis. J. R. Soc. Interface.

[45] Ganesan P, He S, Xu H (2010). Development of an image-based network model of retinal vasculature. Ann. Biomed. Eng..

[46] Lutty GA (2017). Diabetic choroidopathy. Vis. Res..

[47] Lutty GA, McLeod DS, Bhutto IA, Edwards MM, Seddon JM (2020). Choriocapillaris dropout in early age-related macular degeneration. Exp. Eye Res..

[48] Sohn EH (2014). Loss of CD34 expression in aging human choriocapillaris endothelial cells. PLoS ONE.

[49] Mullins RF, Johnson MN, Faidley EA, Skeie JM, Huang J (2011). Choriocapillaris vascular dropout related to density of drusen in human eyes with early age-related macular degeneration. Investig. Ophthalmol. Vis. Sci..

[50] McLeod DS (2002). Quantifying changes in RPE and choroidal vasculature in eyes with age-related macular degeneration. Investig. Ophthalmol. Vis. Sci..

[51] Ramrattan RS (1994). Morphometric analysis of bruch’s membrane, the choriocapillaris, and the choroid in aging. Investig. Ophthalmol. Vis. Sci..

[52] Cheung CMG, Teo KYC, Spaide RF (2021). Pulsatile filling of dilated choroidal vessels in macular watershed zones. Retina.

[53] Yoneya S, Tso MO (1987). Angioarchitecture of the human choroid. Arch. Ophthalmol..

[54] An S, Yu HW, Yao J (2017). GPU-accelerated volumetric lattice Boltzmann method for porous media flow. J. Petrol. Sci. Eng..

[55] Wang Z, Zhao Y, Sawchuck AP, Dalsing MC, Yu HW (2015). GPU acceleration of volumetric lattice Boltzmann method for patient-specific computational hemodynamics. Comput. Fluids.

